# Autoimmune Limbic Encephalitis in a Patient with Acute Encephalopathy and Hyponatremia

**DOI:** 10.1155/2019/9051738

**Published:** 2019-10-07

**Authors:** K. A. Agarwal, M. Albertolle, M. Tiru

**Affiliations:** ^1^Beth Israel Deaconess Medical Center, 330 Brookline Ave., Boston, MA 02215, USA; ^2^Baystate Medical Center, 759 Chestnut Street, Springfield, MA 01199, USA

## Abstract

Acute encephalopathy is a common clinical presentation for hospital admissions. Autoimmune encephalitis is a rare cause of encephalopathy which has increasingly been recognized over the last decade. The detection of various neuronal antibodies has helped diagnose these syndromes, but they have limited availability, mostly in the developed countries. We present a case of a middle-aged female presenting with memory impairment, gait disturbances, and hyponatremia. A clinical diagnosis of autoimmune limbic encephalitis was made based on faciobrachial dystonic seizures, SIADH, and MRI changes 10 days prior to autoantibody titer returned. Prompt treatment with steroids and intravenous immunoglobulin was started with improvement in her neurological symptoms. This case highlights the importance of considering autoimmune encephalitis syndromes in the differential diagnosis of patients with classical neurological presentations and prompt diagnosis and immunotherapy to improve neurological outcomes.

## 1. Introduction

Encephalopathy is a broad term encompassing any abnormality of brain function. This can include deficits in cognition, memory, attention, concentration, or even a general state of consciousness. Etiologies include limbic encephalitis which is a result of autoimmune-induced inflammation of the limbic system, which encompasses the thalamus, amygdala, hypothalamus, and mammillary bodies and is critical in memory, behavior, and emotion. Limbic encephalitis presents most commonly with memory loss with onset over the course of weeks to months. Other symptoms include confusion, seizures, and psychogenic features such as anxiety, depression, hallucinations, and personality changes [1]. Limbic encephalitis can be caused by autoimmune process, which may be paraneoplastic or nonparaneoplastic. Paraneoplastic limbic encephalitis is usually associated with small cell lung cancer, testicular tumors, and thymomas [2], whereas nonparaneoplastic limbic encephalitis has been attributed to antibodies against different channel proteins or receptors in the nervous system. Characterization of these antibodies is relatively recent, with anti-voltage-gated potassium channel (VGKC) antibodies being reported in 2001 [3]. Unfortunately, these specialized antibody assays are only available in some research laboratories, and therefore diagnosis is often missed or delayed when finally made. We present a case of a middle-aged female who was brought in by her family for acute onset memory deficits and ataxia, complicated by in-hospital seizures, with the only finding of syndrome of inappropriate antidiuretic hormone (SIADH) on initial evaluation which did not improve despite correction of sodium levels. She met clinical criteria for limbic encephalitis and received immunotherapy only to be later diagnosed with anti-VGKC limbic encephalitis.

## 2. Case Presentation

**Figure 1:**
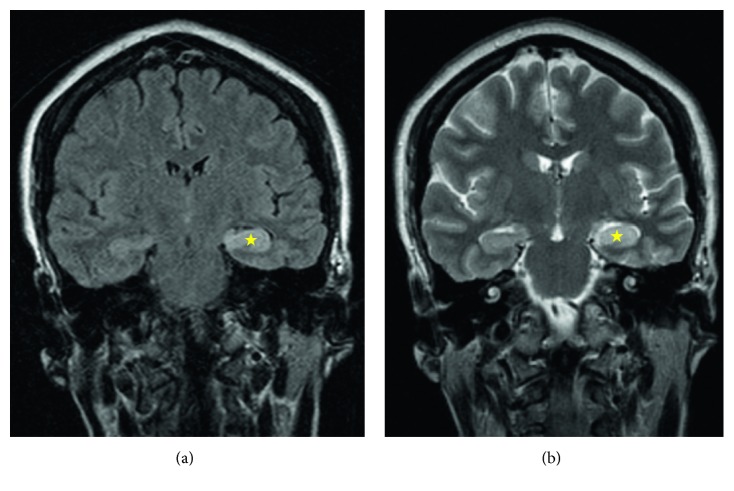
MRI brain with contrast on admission showing a hyperintense left hippocampus with mild swelling and contrast enhancement on (a) FLAIR sequence coronal section and (b) T2-weighted coronal section.

A 47-year-old female with a known medical history of rheumatoid arthritis and chronic pain syndrome was brought to the hospital by her family for a 2-week history of intermittent episodes of confusion, short-term memory loss, slurred speech, increased somnolence, and gait instability. A complete review of systems was also positive for intermittent dizziness, minor falls, and pain at multiple locations. Her medications included methotrexate, golimumab, folic acid, tramadol, and diclofenac as needed for pain. Family history was significant for coronary artery disease in her father. She worked as a cashier and was a lifetime nonsmoker and denied alcohol and illicit drug use. Initial physical exam revealed good personal hygiene, normal vital signs, mild swelling of both her wrist joints without any bony deformities, and an unremarkable heart, lung, and abdominal exam. No rash on skin exam. Neurological examination was significant for 5/5 motor strength in her extremities except limitation at the left wrist because of pain, normal and symmetrical sensations to light touch, vibration, and pinprick, 2+/4 symmetric biceps, patellar and ankle jerks, and absence of any tremors, asterixis, or clonus. Mental status exam revealed normal level of alertness, cooperative behavior, good eye contact, flat affect, intermittent slurring of speech, circumstantial thought process, and poor insight and a score of 22/30 on a Folstein Mini-Mental Status Exam (2/3 immediate word recall, 3/5 backward counting from 100, 3/5 spelling “WORLD” backward, and 0/3 delayed recall). Initial laboratory investigations were unremarkable except hyponatremia (details in Table 1). A serum pregnancy test was negative. CT scan of the head without contrast did not reveal any evidence of intracranial hemorrhage. An MRI of the brain and cervical spine with and without contrast did not reveal any obvious abnormalities except minimal cervical spondylosis at C4–C6 levels. The patient was admitted for altered mental status in the setting of syndrome of inappropriate antidiuretic hormone (SIADH) and started on fluid restriction and eventually urea tablets. Her methotrexate, golimumab, and diclofenac were held. On hospital day 2, the patient was noted to have twitching of her right upper extremity without any other focal neurological deficits. She also did not remember the events of the prior day. Neurology service was consulted; the patient was loaded with levetiracetam and placed on a 48-hour video EEG monitoring. Twelve hours into EEG monitoring, she was noted to have left-sided periodic lateralized epileptiform discharges (PLEDs) and generalized slowing of her background waves. A lumbar puncture was performed and was remarkable for minimally elevated opening pressure and a high IgG index (Table 1). Due to lack of improvement in clinical status and EEG findings of PLEDs, a brain MRI was repeated with coronal sections and revealed T2 hyperintensity with mild swelling of the left hippocampus with abnormal contrast enhancement (Figure 1). Given these findings, there was concern for an inflammatory etiology for her neurological abnormalities, and therefore she was started on 1 g methylprednisolone daily for a suspected autoimmune encephalitis. A CT scan of her head, neck, chest, abdomen, and pelvis was performed next to look for primary tumors that could be causing a paraneoplastic encephalitis, but these were negative for any abnormalities. The patient failed to improve over the next 2 days and was switched to intravenous immunoglobulin (IVIg) at a dose of 400 mg/kg/day for 5 days. Sodium level improved with urea and remained stable between 132 and 136 mmol/L. A repeat video EEG did not reveal any seizures or epileptiform activities this time but did show mild to moderate slowing of the background. At this time, the patient was noted to be walking around the unit without ataxia and her memory had improved mildly. She was able to recall 3/3 objects immediately and 2/3 after 15 minutes. The patient was discharged with close outpatient neurology follow-up. The serum paraneoplastic panel sent early in admission resulted positive for antibody to the neuronal voltage-gated potassium channel (VGKC) 3 days after her discharge, with a titer of 760 pmol/L. Therefore, a retrospective diagnosis of anti-VGKC limbic encephalitis was made. Antibodies to leucine-rich glioma-inactivated protein-1 (anti-LGl1) and contactin-associated protein-2 (Caspr2) were not tested. Anti-NMDA receptor and anti-GAD antibodies were negative.

## 3. Follow-Up/Outcome

**Figure 2:**
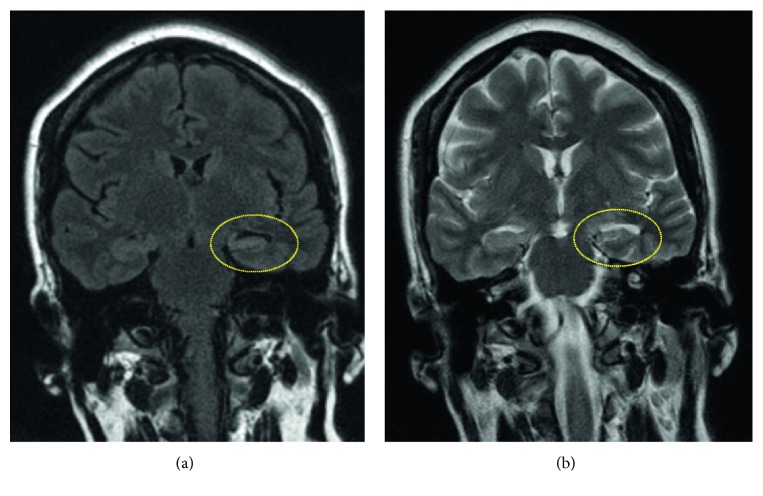
Follow-up MRI brain with contrast, 1 month after discharge, showing improved swelling of the left hippocampus with mild atrophy and volume loss on (a) FLAIR sequence coronal section and (b) T2-weighted coronal section.

Patient returned to the hospital 1 month after discharge with recurrent seizures, short-term memory loss, and ataxia. Repeat MRI brain showed mild volume loss of her left hippocampus (Figure 2), and serum studies revealed persistence of low-titer anti-VGKC antibodies (109 pmol/L). She was treated with a 5-day course of plasmapheresis followed by improvement in her neurological status and discharged home with outpatient neurology follow-up. Unfortunately, she continued to have intermittent memory disturbances over the next 2 months and is currently being managed on an outpatient basis with levetiracetam titrated to a maximum dose of 1500 mg twice daily. She will continue to follow-up with Neurology every month.

## 4. Discussion

Alteration in mental status is a common patient presentation, and the differential diagnosis includes primary neurological as well as secondary causes like metabolic, toxic, hypoxic, vascular, psychiatric, and infectious. Basic evaluation includes cardiac monitoring, measuring serum glucose, electrolytes, alcohol and drug levels, arterial blood gas, thyroid function tests, urine analysis with toxicology, lumbar puncture, and imaging studies. Autoimmune encephalitis, once considered rare, is now being diagnosed more frequently because of the identification of specific neuronal antibodies. As per a recent study, the incidence of autoimmune encephalitis has increased from 0.4 per 100,000 person-years in 1995–2005 to 1.2 per 100,000 person-years in 2006–2015, mostly attributed to increased detection [4]. The California Encephalitis Project reported that 65% of patients with anti-NMDA receptor antibodies were 18 years of age or younger [5]. Limbic encephalitis is usually a paraneoplastic phenomenon but may also occur without an associated cancer. In fact, the neurologic syndrome may present weeks to months before diagnosis of the primary tumor [6].

Even though serum and cerebrospinal fluid assays for auto-antibodies are now available in a limited number of laboratories mostly in Europe and United States, the diagnosis will often be delayed if reliance is placed solely on antibody testing. Early diagnosis and immunotherapy is paramount to achieve neurological improvement and prevent relapses [2]. Therefore, Graus et al. published guidelines for clinical diagnosis of autoimmune encephalitis, including limbic encephalitis [7]. Diagnosis is usually suspected in patients presenting with subacute onset of short-term memory loss, altered mental status, or psychiatric symptoms. They may also have focal neurological findings, unexplained new seizures, CSF pleocytosis, elevated CSF IgG levels, or even MRI findings suggestive of encephalitis. Typical MRI findings of autoimmune limbic encephalitis have been reported in up to 60% of the patients, and these include abnormal hyperintensity of medial and temporal lobes on T2 sequences, more evident on coronal sections and on fluid attenuation inversion recovery (FLAIR) sequences [2]. EEG findings are usually nonspecific but may be used to unveil subclinical seizures or nonconvulsive status epilepticus.

An autoantibody panel on the serum or CSF may reveal antibodies associated with paraneoplastic phenomenon, like anti-Hu, anti-Ma2, anti-amphiphysin, anti-CV2/CRMP5, anti-NMDA receptor, or predominantly nonparaneoplastic autoantibodies like anti-VGKC and anti-GAD65. Anti-Hu antibodies have shown association with small cell lung cancer in younger patients, whereas young patients with anti-Ma2 antibodies should be evaluated for testicular cancer [2]. Anti-VGKC autoantibodies are associated with peripheral nerve hyperexcitability, Morvan's syndrome, and epilepsy besides limbic encephalitis [8]. More recent studies have shown that anti-VGKC antibodies are actually directed against specific proteins associated with the potassium channels: leucine-rich glioma-inactivated protein-1 (LGl1) and contactin-associated protein-2 (Caspr2) [9]. Some patients have positive anti-VGKC antibodies but are seronegative for LGl1 and Caspr2 autoantibodies. These are not associated with the typical neurological findings as described above. Hyponatremia is commonly seen in anti-VGKC limbic encephalitis, more specifically the anti-LGl1 subtype, and has been postulated as a marker to prompt testing for anti-VGKC antibodies in patients with seizures and faciobrachial movements [10]. The exact cause of hyponatremia is unknown, but the expression of LGl1 in both the brain and kidneys has been cited as a possible mechanism [11]. Unlike SCLC, where hyponatremia has been associated with poorer outcomes [12], a clear prognostic link between limbic encephalitis and hyponatremia has not been shown yet.

Our patient met criteria for definite autoimmune limbic encephalitis [7]: subacute onset of memory deficit, elevated CSF IgG index, bilateral brain abnormalities on brain MRI (Figure 1), EEG with slow-wave activity, and reasonable exclusion of alternative causes. Therefore, she was started on immunotherapy with corticosteroids and eventually switched to IVIg due to poor response. Awaiting her autoimmune antibody results would have delayed the diagnosis by over two weeks, hence putting her at risk of worsening neurological dysfunction. The rarity of this disease has limited the development of specific treatment guidelines, and most decisions are guided by retrospective case studies. Treatment options include corticosteroids, which suppress the overall immune response, and other targeted therapies against autoantibody-mediated neuroinflammation. First-line immunotherapy includes methylprednisolone, IVIg, and plasma exchange to remove the autoantibodies. Second-line therapies include rituximab targeting B cells and plasma cells. Alternative antiproliferative agents like cyclophosphamide and mycophenolate mofetil can be used in refractory cases or when steroid-sparing regimes are desired [13].

Despite immunotherapy, clinical relapses are not uncommon. A case series of 63 patients with anti-LGl1-associated limbic encephalitis showed a 27% rate of relapse over a median follow-up of 39 months [14]. In another series, hippocampal atrophy had developed in 7 out of 9 patients with anti-LGl1 antibodies [15]. Our patient was also noted to have volume loss of her left hippocampus (Figure 2) and persistence of low-titer anti-VGKC antibodies 1 month after discharge, which is consistent with prior reports. There are no guidelines for further management of patients with relapses, and treatment is personalized, usually employing alternative immunosuppressive agents.

## Figures and Tables

**Table 1:** 

Parameter	Result	Reference range
*Blood/serum studies*		
Hemoglobin	12.9 g/dL	11.7–16.5 g/dL
Hematocrit	38.7%	35.7–45.8%
WBC count	10.5 k/mm^3^	4–11 k/mm3
Platelet count	382 k/mm^3^	150–460 k/mm^3^
Sodium	127 mmol/L	133–145 mmol/L
Potassium	3.8 mmol/L	3.8–5.2 mmol/L
Chloride	88 mmol/L	98–107 mmol/L
Bicarbonate	27 mmol/L	22–29 mmol/L
Anion gap	12	4–17
BUN	5 mg/dL	6–20 mg/dL
Creatinine	0.6 mg/dL	0.5–1.0 mg/dL
Serum osmolality	257 mOsm/kg	280–290 mOsm/kg
TSH	1.64 IU/L	0.4–4.0 IU/L
Free T4	1.08 ng/dL	0.7–1.8 ng/dL
AST	17 U/L	0–32 U/L
ALT	16 U/L	0–33 U/L
Alkaline phosphatase	86 U/L	35–104 U/L
Total bilirubin	0.5 mg/dL	0–1.2 mg/dL
Vitamin B12	278 pg/mL	232–1245 pg/mL
Calcium	9.4 mg/dL	8.6–10.5 mg/dL
Phosphorous	3.8 mg/dL	2.5–4.5 mg/dL
Magnesium	1.8 mg/dL	1.6–2.3 mg/dL
Glucose	117 mg/dL	70–99 mg/dL
Uric acid	2.8 mg/dL	1.6–7.6 mg/dL
Cortisol (7am)	26 ug/dL	6–18.4 ug/dL

*Urinary studies*		
Urine osmolality	486 mOsm/kg	50–1400 mOsm/kg
Urine sodium	87 mmol/L	20 mmol/L
Urine barbiturate, cannabinoid, cocaine, benzodiazepine, amphetamine, opiate, oxycodone	*Not detected*	*Not detected*

*Cerebrospinal fluid studies*		
Opening pressure	21 cmH20	<20 cmH20
Color	Clear	Clear
WBC	3	0–5
RBC	1	0
Total protein	18 mg/dL	15–60 mg/dL
Glucose	69 mg/dL	50–80 mg/dL
Oligoclonal bands	3	<5
IgG index	0.86	0.30–0.77
Meningoencephalitis PCR panel	*Negative*	*Negative*
CSF gram stain and culture	*Negative*	*Negative*
CSF AFB smear	*Negative*	*Negative*
CSF AFB culture	*Negative*	*Negative*
CSF fungal culture	*Negative*	*Negative*
